# Human Primary Epithelial Cell Models: Promising Tools in the Era of Cystic Fibrosis Personalized Medicine

**DOI:** 10.3389/fphar.2018.01429

**Published:** 2018-12-07

**Authors:** Nikhil T. Awatade, Sharon L. Wong, Chris K. Hewson, Laura K. Fawcett, Anthony Kicic, Adam Jaffe, Shafagh A. Waters

**Affiliations:** ^1^Faculty of Medicine, School of Women's and Children's Health, University of New South Wales, Sydney, NSW, Australia; ^2^Molecular and Integrative Cystic Fibrosis Research Centre (miCF_RC), School of Medical Sciences, Faculty of Medicine, University of New South Wales, Sydney, NSW, Australia; ^3^School of Biotechnology and Biomolecular Sciences, University of New South Wales, Sydney, NSW, Australia; ^4^Department of Respiratory Medicine, Sydney Children's Hospital, Sydney, NSW, Australia; ^5^Centre for Child Health Research, Telethon Kids Institute, The University of Western Australia, Nedlands, WA, Australia; ^6^Occupation and Environment, School of Public Health, Curtin University, Bentley, WA, Australia; ^7^Faculty of Health and Medical Sciences, The University of Western Australia, Nedlands, WA, Australia; ^8^Department of Respiratory and Sleep Medicine, Perth Children's Hospital, Nedlands, WA, Australia; ^9^Centre for Cell Therapy and Regenerative Medicine, School of Medicine and Pharmacology, The University of Western Australia, Nedlands, WA, Australia

**Keywords:** cystic fibrosis, organoid, personalized medicine, CFTR, drug development, sweat chloride, CFTR modulator

## Abstract

Cystic fibrosis (CF) is an inherited disorder where individual disease etiology and response to therapeutic intervention is impacted by CF transmembrane regulator (CFTR) mutations and other genetic modifiers. CFTR regulates multiple mechanisms in a diverse range of epithelial tissues. In this Review, we consolidate the latest updates in the development of primary epithelial cellular model systems relevant for CF. We discuss conventional two-dimensional (2-D) airway epithelial cell cultures, the backbone of *in vitro* cellular models to date, as well as improved expansion protocols to overcome finite supply of the cellular source. We highlight a range of strategies for establishment of three dimensional (3-D) airway and intestinal organoid models and evaluate the limitations and potential improvements in each system, focusing on their application in CF. The *in vitro* CFTR functional assays in patient-derived organoids allow for preclinical pharmacotherapy screening to identify responsive patients. It is likely that organoids will be an invaluable preclinical tool to unravel disease mechanisms, design novel treatments, and enable clinicians to provide personalized management for patients with CF.

## Introduction

Cystic Fibrosis (CF; OMIM 219700) is the most common life-limiting, autosomal recessive monogenic disease in Caucasian populations (Riordan, 2008). It is caused by mutations in the gene encoding CF transmembrane conductance regulator (CFTR), an anion channel essential for regulating trans-epithelial secretion of chloride (Cl^−^) and bicarbonate (HCO${}_{3}^{-}$). The loss of CFTR function leads to abnormalities at the mucosal surfaces in multiple exocrine organs including the lungs, pancreas, liver, and intestine. Notably however, progressive lung disease and respiratory failure are the major cause of morbidity and mortality for most patients (Ratjen et al., 2015). While the regulatory role of CFTR in ion and fluid transport is widely known, the exact mechanism of its defect in the development of CF remains debatable. Two competing hypotheses attempt to explain CF pathogenesis; (1) the “high salt” hypothesis postulates that diminished trans-epithelial Cl^−^ conductance associated with CFTR dysfunction results in increased retention of salt at the mucosal surface, which negates the activity of antimicrobial peptides present in the epithelial apical secretions (Zabner et al., 1998; Wine, 1999); (2) the more favorable “low volume” hypothesis suggests the lack of epithelial sodium channel (ENaC) inhibition in the absence of CFTR function causes sodium (Na^+^) hyper-absorption, with accompanying intracellular flux of Cl^−^ and water (Matsui et al., 1998; Wine, 1999). Mucus becomes dehydrated, viscous and sticky which then leads to decreased airway surface liquid (ASL) height, as well as mucus stasis and obstruction (Riordan, 2008). Irrespective of the mechanism of action, impaired mucociliary clearance in the lung and the innate inability to eradicate inhaled pathological agents are hallmarks of CF. Recurrent cycles of lung infection and exacerbations result in chronic inflammatory responses which then lead to structural lung damage, loss of lung function, and ultimately respiratory insufficiency (Ratjen et al., 2015).

Major advances in symptom management have been instrumental in delaying disease progression in CF. Administration of enzyme replacement therapy, nutritional support, mucolytic agents airway clearance techniques, and antibiotics for bacterial lung infection, along with early detection of disease (newborn screening, pathogen surveillance) has dramatically improved life expectancy in CF patients over the last 4 decades. The median age of death is now above 40 years (Keogh et al., 2018). Importantly, a class of small molecule compounds termed CFTR modulators were recently discovered. These compounds directly correct defective CFTR protein in CF patients and have now transformed the therapeutic landscape of CF in a precision-based fashion. The first part of this review outlines the current developments in CFTR modulators addressing the different dysfunctional CFTR mutation class and highlights the need for individualized strategies to restore their function.

The second part of this review discusses the evolving use of different epithelial cell models and reporter assays for assessing clinical response to CFTR-directed drugs on a personalized basis, and highlights the strengths and limitations of each application. This review also elaborates on the breakthrough discoveries of long-term cultures of patient-derived 3-dimentional (3-D) airway and rectal organoids. We also discuss their emerging use as *ex vivo* biomarkers and preclinical predictive tools for the disease.

## Current CFTR-Based Therapy With CFTR Modulators

More than 2,000 CFTR mutations have been identified so far and at least 336 of these are reported to lead to symptoms characteristic of CF (Cystic Fibrosis Mutations Database report 31 August 2018 (US CF Foundation, 2011). It is thus perceivable that a multi-pronged approach is required to target the different defective mechanisms that each CFTR mutation confers. For the past 15 years, high-throughput screening (HTS) has accelerated the process of drug discovery. To identify candidate CFTR modulators, hundreds of thousands of chemical compounds with diverse structures were screened and the potential of a compound to rescue or activate CFTR was tested in cellular-based assays (Pedemonte et al., 2005; Van Goor et al., 2006; Sutanto et al., 2018). The promising compounds, called “hits,” underwent further medicinal and chemical optimization to improve potency and minimize potential off-target activities of the compounds. This process has led to successful identification of multiple compounds, some of which have moved forward to human clinical trials.

The different approaches targeted toward correcting each CFTR mutation class as well as the compounds currently tested in clinical trials are summarized in Table 1. CFTR-modulating compounds are classified into five main groups: read-through agents, correctors, potentiators, stabilizers, and amplifiers. Of these, two classes of modulators (potentiators and correctors) have gained regulatory approval to treat CF patients with specific CFTR mutations.

**Table 1:** CFTR mutation class and current modulator therapeutic approach.

**Mutation class**	**Molecular defect**	**Type of mutation**	**Therapeutic approach**	**Approved or drugs in clinical trial**	**Mutation examples**
IA^**~**^	No mRNA synthesis	Large deletions	Bypass therapies (Activate alternative Cl^−^ channels)	–	Dele2, 3(21 kb) 1717-1G->A 621+1G->T
IB^**~**^	Protein synthesis	Nonsense (PTC), Frame-shift, Splicing	Read-through compounds	Phase 2: QBW276, SPX-101 Phase 1: AZD5634, BI443651	G542X, W1282X
II	Trafficking	Missense	Correctors and potentiators	Approved: Orkambi, Symdeko Phase 3: VX-445^#^, VX-659^#^ Phase 2: VX-152^#^, VX-440^#^, GLPG2222, GLPG2737, FDL169 Phase 1: PTI-801	F508del, R560T, A561E
III	Channel Gating	Missense	Potentiators	Approved: Ivacaftor Phase 2: VX-561, QBW251, GLPG1837, GLPG2451, GLPG3067 Phase 1: PTI-808	G551D, S1251N, G178R
IV	Conductance	Missense	Potentiators	Approved: Ivacaftor	R334W, R347P, R117H
V	Protein synthesis	Missense, Alternative splicing	Correctors, Potentiators, Antisense Oligonucleotides	Approved: Ivacaftor	3849+10kbC>T, 3272-26A>G, 2789+5G>A
VI	Reduced CFTR stability at PM	Missense, Frameshift	Stabilizers	–	120del23, N287Y, Q1412X

Compounds starting with VX are developed by Vertex Pharmaceuticals, GLPG by Galapagos NV and AbbVie, FDL, Flatley discovery lab; QBW, Novartis pharmaceuticals; PTI, Proteostasis therapeutics; SPX, Spyryx biosciences; AZD, AstraZeneca; BI, Boehringer ingelheim; Orkambi, VX-809+VX-770; Symdeko, VX-661+VX-770; Ivacaftor, VX-770;

# *Triple combination therapy with VX-661 and VX-770*.

*PTC, Premature termination codons; PM, Plasma membrane; ~, classification is based on (Marson et al.,^2016^)*.

### Potentiators

Potentiators were developed for CFTR mutant proteins that are expressed at the apical membrane of epithelial cells but are functionally impaired. HTS performed by Vertex pharmaceuticals involving 280,000 small molecule compounds led to the discovery of the potentiator VX-770 (Generic name: Ivacaftor; Trade name: Kalydeco) (Van Goor et al., 2010). Ivacaftor corrects the gating impairment of Class III mutations. Its use has now been extended beyond the most common Class III G551D-CFTR mutation, to additional Class III mutations, as well as CFTR mutations with conductance (Class IV−R117H) or biosynthesis (Class V−3,849 + 10 kb C>T) defects. In clinical studies of participants with G551D-CFTR mutations, the impact of Ivacaftor on CFTR was evidenced by normalization of sweat Cl^−^, improved lung function (10% mean increase in FEV_1_), reduced episodes of pulmonary exacerbations and improved body mass index (BMI) (Accurso et al., 2010; Ramsey et al., 2011; Davies et al., 2013).

### Correctors

The potentiators' mode of action does not benefit the majority of the CF population who have the Class II F508del-CFTR mutation, as F508del-CFTR is degraded while transitioning through the endoplasmic reticulum, with very little or no mutant protein reaching the apical membrane of epithelial cell. Effective rescue of the F508del mutation thus requires chaperones that can repair defective protein folding and rescue trafficking of the mature CFTR to the plasma membrane. VX-809 (Generic name: Lumacaftor; available as Lumacaftor-Ivacaftor combination therapy—Trade name Orkambi) restored F508del-CFTR channel activity to 15% of wild-type CFTR in *in vitro* preclinical testing performed in primary airway epithelial cells (Van Goor et al., 2012). This result led to Lumacaftor monotherapy clinical trials in CF patients homozygous for the F508del-CFTR mutation, where a significant improvement in sweat Cl^−^ concentrations were observed but lung function remained unchanged (Clancy et al., 2017). Considering that F508del channel gating defect, administration of Lumacaftor-Ivacaftor combination therapy was proposed as a solution that may augment correction of CFTR function to clinically significant levels.

### Combination Therapy

Lumacaftor-Ivacaftor combination therapy increased CFTR activity at the plasma membrane *in vitro* (Van Goor et al., 2012). However, results from phase III trials in children and adults homozygous for the F508del-CFTR mutation showed that the combination therapy failed to produce the magnitude of clinical improvements observed with Ivacaftor. While a reduction in pulmonary exacerbations and improved BMI was observed, there was only a modest improvement in lung function (2–3%) (Boyle et al., 2014; Wainwright et al., 2015). In addition, CF patients receiving Lumacaftor-Ivacaftor combination therapy reported unwanted side effects such as dyspnoea, liver damage, and bronchospasm. Lumacaftor is also associated with significant drug-drug interactions which alter its pharmacokinetic profile and potentially hamper its therapeutic efficacy (Talamo Guevara and McColley, 2017). Tezacaftor is a new CFTR corrector with an improved pharmacokinetic profile, longer half-life and less drug-drug interactions compared to Lumacaftor. It has recently been approved as a combination therapy with Ivacaftor (Trade name: Symdeko/Symveki) for the treatment of patients homozygous for F508del mutation by the Food and Drug Administration (FDA) and European Medicines Agency. Phase III clinical trials data showed that improvement in lung function with Tezacaftor-Ivacaftor combination therapy was generally comparable or better than those observed in patients treated with Lumacaftor-Ivacaftor combination therapy (Rowe et al., 2017; Taylor-Cousar et al., 2017). It is notable however that while overall benefit was demonstrated, individual patient responses have been heterogeneous in the clinical trials of both Lumacaftor-Ivacaftor and Tezacaftor-Ivacaftor.

### Theratyping

It is now known that patients display a spectrum of responses to CFTR-modulator drugs despite having the same CFTR mutation variant (Wainwright et al., 2015; Donaldson et al., 2018). This suggests that although the current classification system may be an important indicator for prognosis and disease severity in CF, it is inadequate for predicting how individual patients respond to therapy. Indeed, *in vitro* experimental studies have shown that contrary to findings in the Class II F508del-CFTR mutation, other Class II processing mutations such as N1303K, R560S, and G85E could not be rescued by Lumacaftor treatment (Awatade et al., 2015, 2018; Dekkers et al., 2016b; Lopes-Pacheco et al., 2017). These results suggest that the underlying pathomechanism of each CFTR mutation is distinct and that individualized strategies to restore their function may be warranted.

To address this, CFTR mutations have been classified according to their response to modulator compounds. This approach termed “theratyping,” groups together patients who harbor different CFTR mutations but respond to the same CFTR-directed compounds. It is clear that treatment regimens of the future will need to take into consideration the individual's genetic makeup and not just their CFTR mutations. A personalized approach will optimize patient's clinical outcomes by accounting for the specific genetic mutations of the individual patient.

## Toward Personalized Modulator Therapy

One of the major hurdles to the development of novel treatment regimens in CF is the bench-to-bedside translation of scientific knowledge. Many drugs that perform well in a laboratory setting fail to advance in clinical trials, largely due to inappropriate selection of *in vivo* and *in vitro* models for HTS. In the context of CF, animal models and immortalized epithelial cell lines do not fully portray patient-specific disease phenotypes. Animal models of CF have provided insights into disease pathophysiology. However, their generation is time consuming, expensive, and more importantly they poorly represent the full repertoire of disease manifestations in individual patients. For instance, mouse models of CF do not have lung disease and bacterial infections, attributed to the compensatory effect of a secondary Cl^−^ ion channel (Lavelle et al., 2016). On the other hand, immortalized epithelial cell lines derived from primary patient material have contributed tremendously to CF research, but not without limitations. Their generation from primary patient material is very inefficient. It involves extensive adaptation and selection to *in vitro* 2-D culture conditions, as only rare clones are able to expand and be maintained over many passages. Furthermore, these cell lines may have undergone substantial genetic changes and no longer retain features of the original parental cells. Drug development pipeline for new CFTR-directed compounds have relied heavily on unpolarized Fischer rat thyroid (FRT) epithelial cell lines. It is thus not surprising that this model has a higher propensity of false-positive and false-negative “hits” compared to that performed in primary human bronchial epithelial (HBE) cells.

## Patient-Derived Organotypic Cellular Models for Personalized Medicine

Although CF is a monogenic disease, the diverse mutation variants identified within the CFTR gene as well as the presence of modifier genes (known and unknown), warrants adoption of new technologies to extend research capabilities. There is a clear unmet need for a representative library of patient-specific epithelial cell models for disease modeling, preclinical testing of drug response, and biobanking for future drug discovery. The cell models can be derived from embryonic stem cells (ESCs), induced pluripotent stem cells (iPSCs), or tissue-resident adult stem cells. Whereas, ethical concerns pertaining the source of human ESC, limit their use in research, generation of CFTR gene corrected iPSC lines enable disease modeling, drug discovery and toxicology studies [reviewed in (Pollard and Pollard, 2018)]. In this Review, we focus on airway and intestine epithelium models derived from adult stem cells.

### Human Airway Epithelial (HAE) Cells

The pulmonary epithelium is divided into three regions; upper (nasal and oral cavities), lower (trachea and primary bronchi), and distal small airway epithelia (alveolar). Persistent inflammation, bacterial colonization, and airway structural changes in CF occur in the lower respiratory tract. Primary HBE cells are therefore the gold standard for studying CF disease pathogenesis and evaluating CFTR functional response (Van Goor et al., 2006; Awatade et al., 2015). HBE cells can be isolated from biopsy samples, lung explants and cadavers. Explanted lung and post-mortem samples from individuals with CF provide high cell yield. However, the extensive damage to the tissue, particularly the epithelial cell layer, plus the presence of chronic microbial colonization present technical challenges to establishing successful *ex vivo* cultures. Acquisition of airway biopsies or brushings involves highly invasive procedures, while bronchoalveolar lavage (BAL) fluid and induced sputum samples usually do not provide sufficient cell counts to initiate culture. Therefore, supply of CF patient-derived HBE cells are often limited and hard to come by.

Human nasal epithelial (HNE) cells are increasingly used as surrogates for the lower airway epithelium in CF research (de Courcey et al., 2012; Brewington et al., 2018a). HNE cells demonstrate many characteristics common to HBE cells including the ability to form polarized, pseudostratified epithelium mimicking *in vivo* airways and the expression of ion channels such as CFTR. Their advantage is the lack of need for invasive procurement (Clarke et al., 2013). HNE cells are grown using the same culture media and protocol as for HBE cells.

#### Human Airway Epithelial (HAE) Cell Culture Models

Primary human airway epithelial cells are conventionally cultured as monolayers (2-D cultures). Expansion of epithelial cells is often necessary in the initial passages for biobanking purposes and to generate enough cell numbers needed to differentiate cells under air-liquid interface (ALI) conditions. HBE cells are most “*in vivo*” like when fully differentiated; they display a striking phenotypic resemblance to the lower airway epithelium. They form pseudostratified epithelium with mucociliary differentiation indicated by the presence of functional beating cilia and mucus secretion. They also exhibit characteristic epithelial barrier functions, including expression of cell junctional proteins and the development of robust trans-epithelial electrical resistance (TEER) values (Berube et al., 2010). The polarized, differentiated phenotype is critical for *in vitro* measurement of CFTR function as the protein is primarily expressed at the apical surface of ciliated cells. While ALI cultures accurately represent *in vivo* phenotypes, their wider use is deterred by limited propagation of cells in culture, attributed to squamous transformation and cellular senescence (Gentzsch et al., 2017). To date, three expansion methods have been used to extend the lifespan of cells and delay cellular senescence beyond that of the standard cultures.

#### Improved Cell Expansion Culture Methods

Conditionally reprogrammed cells (CRCs) are generated by co-culturing patient-derived airway epithelial cells with irradiated fibroblast feeder cells. Specialized conditioned media (termed F-Media) which contains Rho-associated protein kinase (ROCK) inhibitor, promotes serial passages of airway epithelial cells and enhances population doubling without compromising the characteristic epithelial cell morphology. Both ROCK inhibitor and the feeder layer are essential in maintaining the stem cell-like phenotype evident in CRCs (Reynolds et al., 2016; Martinovich et al., 2017). Their removal results in differentiated cell lines, with intact barrier function and the ability to polarize and form mucociliary epithelium. CFTR-mediated Cl^−^ transport in these cells is also preserved.

A modified CRCs protocol using BEGM, ROCK inhibitor and an irradiated feeder layer cultured in reduced oxygen concentration (2%) demonstrated some advantage over the standard CRCs. The modified CRCs could support airway epithelial cell growth up to passage 10 with robust formation of pseudostratified epithelium at the extended passage, although a modest decrease in CFTR-dependent Cl^−^ transport was observed. Meanwhile, reduced numbers of ciliated cells and goblet cells were observed in standard CRCs cultures at P10 and robust TEER could not be established (Peters-Hall et al., 2018).

A feeder-free culture protocol that relies on disruption of SMAD signaling pathway through inhibition of dual ligands, transforming growth factor-beta (TGF-β) and bone morphogenic protein (BMP), showed airway epithelial cells could be expanded up to 18–25 passages without loss of proliferative potential (Mou et al., 2016). Combined TGF-β/BMP inhibition led to basal cell hyperplasia with hyper-proliferative p63 cells (basal cell marker) and produced homogenous, tightly packed cells, resembling stem cell morphology. Epithelial cells expanded using dual SMAD inhibition method can undergo mucociliary differentiation up to P12 with preserved TEER although Na^+^ currents and Cl^−^ conductance declined fairly rapidly after serial passage 6 (Mou et al., 2016).

### Three-Dimensional Airway Spheroids and Organoids

Two-D cultures lack a third dimension, the scaffolding extracellular matrix (ECM), which establishes intercellular cues or network in the *in vivo* airway epithelium. Therefore, 3-D cell cultures are a major area of development, where cells are cultured in a matrix (such as matrigel) or are cultured in such a manner that they develop ECM-like scaffolds between them, thus mimicking the *in vivo* phenotype more faithfully. While cell-derived ECMs such as Matrigel have been most commonly used for development of organoids, their undefined components introduce inconsistency in replicating the native 3-D culture environment (Czerwinski and Spence, 2017). To overcome this challenge, new biomaterial systems, such as polymers and hydrogels are being developed [reviewed in (Dye et al., 2017)].

Different methods for generating organoids (or spheroids) from human lung cells have been described to date, each producing organoids with distinct definitive structure and cellular compositions. Barkauskas and colleagues have elegantly reviewed the different lung organoids (airway organoids inclusive) established from varying lung progenitor cell populations, including basal cells in the proximal airways, secretory club Clara cells in bronchioles and alveolar type II cells in the alveoli, as well as those from embryonic and iPSCs (Barkauskas et al., 2017). Given CFTR is primarily expressed at the apical surface of ciliated cells and recently discovered in the pulmonary ionocytes in tracheal epithelium (Montoro et al., 2018; Plasschaert et al., 2018), only airway organoids displaying proximal differentiation are discussed here in light of their relevance for measuring CFTR functional response (Table 2).

**Table 2:** Comparison of three-dimensional organotypic airway epithelial culture methods.

	**Respiratory—nasal and bronchial**		
	**Airway explant spheroid**	**Airway basal cell spheroid**	**Long term expanding airway organoid**
		**Method 1—Dome Method 2–25% layer**	
Cultures	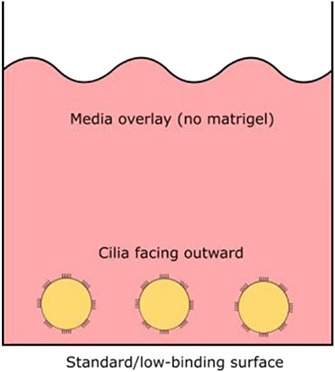	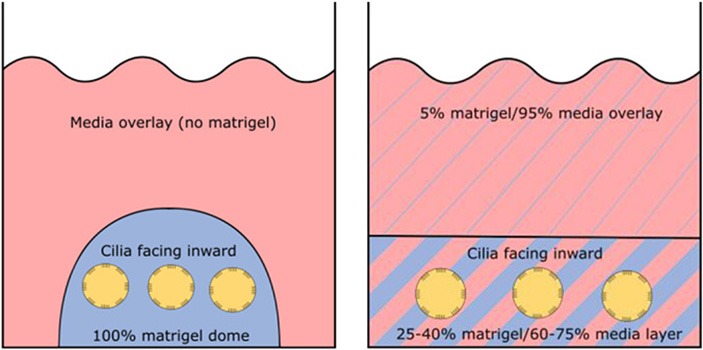	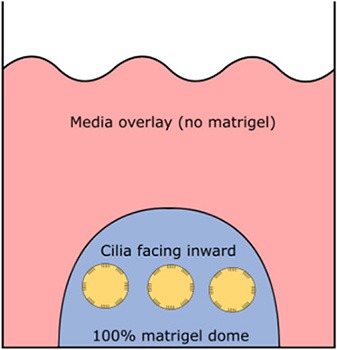
Cellular source	Freshly isolated non-dissociated airway epithelial sheets	Airway basal progenitor cells expanded as traditional primary cultures or conditionally reprogrammed cultures	Lung biopsies and cells from BAL fluid
Media	Standard, non-differentiating culture media e.g., BEGM	Differentiation media—same as those used for 2D ALI cultures	Media containing biochemical cues for self-renewal. Content: Advanced DMEM/F12 R-Spondin 1 Noggin FGF 7 FGF 10 A83-01 Y-27632 SB202190 B27 supplement N-Acetylcysteine Nicotinamide Glutamax
Require ECM (matrigel)	No	Yes	Yes
Constitute differentiated epithelium structure	Yes (*ex vivo—*already differentiated)	Yes (after 14–21 days culture)	Yes (duration to form not reported)
Orientation of apical ciliated side	Facing outwards (media)	Facing inwards (lumen)	Facing inwards (lumen)
Use of cultures	End-point experiments	End-point experiments	Can be passaged for on-going cultures (reported up to P18)
CFTR functional assay	Yes—spheroids shrink when CFTR is activated	Yes—spheroids swell when CFTR is activated	Yes—spheroids swell when CFTR is activated
Cryopreservation	No	No	Not reported
References	Bridges et al., 1991 Pedersen et al., 1999 Deslee et al., 2007 Guimbellot et al., 2017	Danahay et al., 2015 Hild and Jaffe, 2016 Brewington et al., 2018c	Sachs et al., 2018 Zhou et al., 2018

*2D, Two-dimensional; ALI, Air-liquid interphase; BAL, Bronchoalveolar lavage; BEGM, Bronchial epithelial cell growth media; ECM, Extracellular matrix; FGF, Fibroblast growth factor; TGF-β, Transforming growth factor-beta*.

#### Three-Dimensional Airway Explant Spheroid (ECM—Free)

Airway spheroid cultures were first described in 1991 when nasal epithelial multicellular spheroids were generated from non-dissociated nasal epithelial sheets (Bridges et al., 1991). These explant spheroids were maintained in culture media (no matrigel) and were reported to form rapidly, within 2–5 days. Explant spheroids have an apical-membrane-out orientation; apical ciliated cells face the media bath and the basolateral membrane lines the fluid-filled lumen (Bridges et al., 1991). Pedersen and colleagues later found amiloride-sensitive Na^+^ transport drives changes in the lumen size, suggesting nasal spheroids may be a useful model for ion/fluid transport studies and for investigating diseases associated with defective ion channels such as cystic fibrosis (Pedersen et al., 1999). Nasal spheroids were also responsive to forskolin stimulation (Guimbellot et al., 2017). As the CFTR-expressing apical side of nasal spheroids face outward, forskolin causes the outflow of fluid from the lumen to the exterior bath and the spheroids to shrink in size. This response was blunted in spheroids established from CF patients but could be partially rescued by CFTR modulators (Guimbellot et al., 2017). Notably, successful spheroids have also been established from bronchial brushings, although CFTR function was not tested in the study (Deslee et al., 2007). Interestingly, no evidence of cell proliferation was observed throughout the nasal spheroid formation process and the spheroids plateaued in size once fully formed. This suggests that the spheroids are self-organizing aggregates of terminally differentiated epithelial cells only (Castillon et al., 2002). While spheroids may be ready for *ex vivo* CFTR measurement rapidly, they are short-lived, lasting for up to 12 weeks only and biobanking for future drug screening is not possible (Guimbellot et al., 2017).

#### Three-Dimensional Spheroid From Airway Basal Progenitor Cells

Airway spheroids, both bronchial and nasal can also be derived from primary airway basal cells embedded in the basement membrane matrix matrigel (Hild and Jaffe, 2016; Brewington et al., 2018c). Matrigel is fundamental for the formation of intact spheroids, with a lumen surrounded by a slightly thickened wall upon maturity (7–14 days) and a complete cell-apex-in morphology (Danahay et al., 2015; Brewington et al., 2018c). Different culture protocols using varying concentrations of matrigel have been reported; (1) cells are resuspended in 100% matrigel and seeded as spherical drops, then overlaid with ALI differentiation media; (2) cells are resuspended in 5% matrigel with ALI differentiation media and then layered over a denser 25–40% matrigel base layer which the cells sink into (Hild and Jaffe, 2016; Tan et al., 2017; Brewington et al., 2018c). Method 1 (100% matrigel) was reported to yield the best success, as the sinking method could result in formation of some spheroids with cell-apex-out morphology and disorganized cellular aggregates (Brewington et al., 2018b), likely due to incomplete embedding of cells in the denser matrigel base layer e.g., growing on top of or at the interface of the denser matrigel base layer. For certain downstream applications such as live imaging (e.g., forskolin-induced swelling—see section The Cell Model of the Future), the sinking method confers an advantage because spheroids are suspended in the same plane and permit HTS. We also note that successful differentiated, pseudostratified spheroid formation has been reported from airway basal cells maintained in the standard BEGM media and those derived from conditionally reprogrammed culture (Hild and Jaffe, 2016; Brewington et al., 2018c). Airway basal cell spheroids have no self-renewal capacity and are used for end-point applications.

#### Three-Dimensional Airway Organoid—Long-Term Expanding

Long term expanding human airway organoids were first reported by Sachs et al. (Sachs et al., 2018). These organoids were established from lung biopsies or cells isolated from BAL fluid and then cultured in media containing biochemical cues for self-renewal such as R-spondin, Noggin, fibroblast growth factor (FGF) and TGF-β inhibitors. The established organoids comprised of a polarized, pseudostratified airway epithelium containing basal cells, ciliated cells, mucus-producing goblet cells, and secretory club (Clara) cells. The airway organoids are amenable to passaging by mechanical disruption every other week for at least a year, with no loss in proliferation reported up to P18. In addition, single cell suspensions dissociated from the indefinitely expanding airway organoids yield an improved 2-D ALI culture (Zhou et al., 2018). These cultures displayed full ALI differentiation by day 12–14 as opposed to 21–28 days for conventional cultures grown from 2-D basal cells (Zhou et al., 2018). Together, these findings suggest the possibility of expanding isolated primary lung epithelial cells in 3-D, given cells maintained under conditionally reprogrammed and dual SMAD inhibition culture conditions do not expand beyond P10 without reaching senescence (Mou et al., 2016; Gentzsch et al., 2017). The indefinite expansion of airway organoids also means in theory the availability of “endless” amounts of ready-to-use airway epithelial cells.

### Human Intestinal Epithelial Cells

While work in CF research has primarily focused on primary airway epithelial cells (the gold standard), patient-derived intestinal epithelial cells also present an invaluable resource in characterizing the relationship between the CFTR gene mutation and the disease phenotype. More recently, they play an important role in the development of HTS strategies to elucidate novel drugs for the treatment of CF.

#### Intestinal Organoids

Compared to human airway tissue, colon tissue damage in CF patients is minimal and the rectal epithelium is accessible in a less invasive manner. The abundant expression of CFTR in the distal colon makes rectal biopsies an attractive cellular source for interrogating CFTR function (Hug et al., 2011). Assessment of intestinal current measurement (ICM) as a readout for CFTR activity has provided strong evidence for the diagnostic and prognostic utility of rectal biopsies (De Jonge et al., 2004; Hirtz et al., 2004; Mall et al., 2004; De Boeck et al., 2006; Taylor et al., 2009; Derichs et al., 2010; Sousa et al., 2012; Clancy et al., 2013; Cohen-Cymberknoh et al., 2013). ICM can be applied to biopsies collected from CF patients which are subsequently exposed to CFTR modulators, to assess the efficacy of treatment in a personalized manner. One concern is the possibility of reduced penetration of modulator drug into the biopsy tissues under *ex vivo* conditions. Other limitations to the use of rectal biopsies include the small number of biopsies collected (4–8 biopsies) and the need for all biopsies to be tested on collection day i.e., the biopsies cannot be preserved for further testing.

Emergence of intestinal organoids (or mini-guts) overcome these limitations by extending the use of rectal biopsies in cultures. Intestinal organoids can be grown from crypts isolated from freshly isolated rectal biopsies. Crypts are rich in Lgr5^+^ stem cells which grow and differentiate into self-organizing, multicellular structures (Sato et al., 2009; Jung et al., 2011). These organoids contain all of the distinct intestinal cell types present in the *in vivo* epithelium (Leushacke and Barker, 2014). The growth and differentiation of Lgr5^+^ stem cells into eventual closed epithelial structures with an internal lumen requires a fine balance of growth factors (R-spondin, EGF, Noggin, Wnt-3a), inhibitors of TGF-β and BMP signaling and the basement membrane matrix (matrigel). Intestinal organoids can be indefinitely cultured and remain genetically and phenotypically stable upon repeated passaging and long term culture (Ikpa et al., 2014). Capitalizing on the high expression of CFTR in rectal tissues and rapidly expanding stem cells, they make an attractive model for assessment of CFTR functional response in pharmacologic testing. They provide the added advantage to test combination of modulators without established safety profiles as part of pre-clinical evaluation in CF patients with rare CFTR genotype.

## Forskolin-Induced Swelling Assay as a Proxy for CFTR Function

The primary functional assay to assess CFTR activity in organoids, the forskolin-induced swelling (FIS) assay is CFTR dependent. This approach does not measure the net transepithelial ionic transport. Rather, forskolin is used to stimulate intracellular cAMP production which then activates CFTR at the plasma membrane. CFTR activation drives chloride and fluid flux to the organoid lumen (apical membrane facing inwards), causing rapid swelling of organoids with functional CFTR or those derived from non-CF subjects. This swelling effect is significantly reduced or absent in organoids derived from CF subjects who exhibit differing rates of FIS with different classes of CFTR mutations and also between individuals with the same CFTR mutation (Dekkers et al., 2013). There is accumulating evidence that the CFTR modulator-corrected FIS response is predictive of patient-specific clinical response, with close correlation observed between rescued swelling and improvement in lung function (measured by FEV1) and sweat chloride concentration (Dekkers et al., 2016a; Guimbellot et al., 2017; Brewington et al., 2018c). Intestinal organoids can be disrupted to single cells to generate 2-D-monolayers on porous membranes for electrophysiological studies. FIS was shown to positively correlate with forskolin-induced current in subject-matched organoid-derived monolayers, supporting the notion that CFTR-dependent fluid secretion in rectal organoids reflects CFTR-dependent ion transport (Zomer-van Ommen et al., 2018)

The FIS assay has been adapted for use in airway organoids but is much less well-characterized compared to rectal organoids (Guimbellot et al., 2017; Brewington et al., 2018c; McCarthy et al., 2018). Studies performed thus far, have involved cultures from small datasets of patients. Given the lack of standardized culture protocol for airway organoids, forskolin induces either swelling or shrinking of airway organoid depending on the orientation of CFTR-expressing apical epithelium (swelling for apical membrane facing inwards organoids and vice versa). Similar to rectal organoids, forskolin-induced changes in cross sectional area is dependent on the severity of CFTR genotypes, and could be altered with CFTR modulators. However, the changes are far smaller given the lower expression of CFTR in the airways compare to the intestine. It is noteworthy that only a single concentration of forskolin (10 μM) has been interrogated in airway organoids FIS, while a total of eight concentrations (0.008–5 μM) were used in rectal organoids (Dekkers et al., 2016a; Guimbellot et al., 2017; Brewington et al., 2018c). To detect CFTR rescue with adequate resolution and sensitivity, higher-powered objectives are used resulting in lower throughput application compared to rectal (Dekkers et al., 2016a; Guimbellot et al., 2017; Brewington et al., 2018c). Establishment of a broader assay dynamic range may facilitate higher throughput use of FIS in airway organoids. Although preliminary, favorable correlation between *in vitro* FIS response to clinical response FEV1, sweat chloride concentration and BMI have been reported (Brewington et al., 2018c; McCarthy et al., 2018).

## The Cell Model of the Future

In this Review, we described the rapidly developing field of organoid models specific to CF. Organoids, with their close resemblance to the human organs most affected by CF disease, hold great appeal for translational research. The capacity to adapt these models for assays such as FIS is important for theratyping and for conferring decisions on personalized CFTR pharmacotherapy. This provides an almost immediate application of *in vitro* research findings in the clinical setting. Nonetheless, it is apparent that each model has respective strengths and limitations (Table 3). The question as to which model has the essential features for precision medicine in CF and best predicts the long-term clinical benefits of a drug remains. Is it sufficient to use just one model and if so which cell model is best?

**Table 3:** Comparison of human cell models relevant for CF therapeutic application.

**Cell models**	**Established immortalized cell lines**	**Pulmonary**				**Gut**		
		**HBE**		**Nasal**		**Intestinal**		
		**Bronchial ALI**	**Bronchial organoid**	**Nasal ALI**	**Nasal organoid**	**Rectal biopsies**	**Rectal organoid**	**Organoid 2-D monolayer**
Patient-specific	No	Yes	Yes	Yes	Yes	Yes	Yes	Yes
Tissue source	-	Lung explants Bronchial brushing/biopsies Bronchoalveolar lavage fluid		Nasal brushing/curettage		Rectal biopsies		
Invasiveness of sample acquisition	-	+++		+		++		
Easy-to-culture	- Flexible - Easy to manipulate	- Limited expansion and differentiation potential (decreases as a function of passage)				- Long term expanding cultures		
		- ALI cultures take 4 weeks to form pseudostratified epithelium -No standardized protocol available for airway organoids				- Long term expanding cultures -Established and standardized protocol for organoid -Monolayer in development phase		
Phenocopies CF lung	No (prone to artifact)	Yes (gold standard)	Yes	Yes	Yes	No	No	No
Phenocopies CF intestine	No	No	No	No	No	Yes	Yes	Yes
CFTR functional readout	- Ussing - Halide assay	- Ussing	- FIS	- Ussing	- FIS	ICM	- FIS/SLA	Ussing
Assay dynamic range	Large	Moderate	Moderate	Small	Small	Large	Large	Large
Biochemical/physiological readout	- CFTR western blotting	- CFTR western blotting - ASL -CBF	- CFTR western blotting	- CFTR western blotting	- CFTR western blotting		- CFTR western blotting	
Drug screening	- High-throughput - Hit-to-lead drug development	- Mid-throughput - Checkpoint before drugs enter clinical trial/use	Exploratory	Mid-throughput	Exploratory	Low-throughput	Mid-throughput	Exploratory
Predictive value	++	+++	Exploratory	Exploratory	Exploratory	++	++	Exploratory

*+++, High; ++, Moderate; +, Low; 2-D, 2-Dimensional; ALI, Air-liquid interphase; ASL, Airway surface liquid; CBF, Cilia beating frequency; FIS, Forskolin-induced swelling; HBE, Human bronchial epithelial; ICM, Intestinal current measurement; SLA, Steady-state lumen area*.

Intestinal organoids are the most developed so far amongst the 3-D model systems and seemingly an easier model to establish. But how representative is the intestinal epithelium to its respiratory counterpart? There are clear physiological differences between the airway mucosal surface and the gut. First, alternative ion channels critical for solute and water transport in the airways such as ENaC and calcium-activated chloride channels (CaCC), are either absent or present in negligible amounts in the gut to be functional (Rajendran et al., 2018). A modified swelling assay showed airway but not rectal organoids swell upon addition of E_act_, an activator of the alternative chloride channel TMEM16A (Sachs et al., 2018). Second, there is no mucociliary clearance in the gastrointestinal tract. These differences highlight the need for organoids of bronchial or nasal epithelial origin to provide a closer resemblance to the *in vivo* airways. It then seems logical that one will need to choose the most appropriate 3-D cell culture model for each specific application.

## Author Contributions

SAW, NTA, and SLW wrote the manuscript with critical input from CKH, LKF, AK and AJ.

### Conflict of Interest Statement

AK is a scientific advisor for Unizyme Laboratories A/S. The remaining authors declare that the research was conducted in the absence of any commercial or financial relationships that could be construed as a potential conflict of interest.

## Abbreviations

2-D: 2-Dimensional
3-D: 3-Dimensional
ALI: Air-liquid interface
ASL: Airway surface liquid
BAL: Bronchoalveolar lavage
BEGM: Bronchial epithelial growth media
BMI: Body mass index
BMP: Bone morphogenic protein
CaCC: Calcium-activated chloride channels
CF: Cystic Fibrosis
CFTR: Cystic Fibrosis transmembrane conductance regulator
Cl^−^: Chloride
CRCs: Conditionally reprogrammed cells
DMSO: Dimethyl sulfoxide
ECM: Extracellular matrix
ENaC: Epithelial sodium channel
FEV1: Forced expiratory volume—one second
FIS: Forskolin-induced swelling
FRT: Fischer rat thyroid
HAE: Human airway epithelial
HBE: Human bronchial epithelial
HCO${}_{3}^{-}$: Bicarbonate
HNE: Human nasal epithelial
HTS: High-throughput screening
ICM: Intestinal current measurement
Na^+^: Sodium
P#: Passage #
PTC: Premature termination codon
ROCK: Rho-associated protein kinase
TEER: Trans-epithelial electrical resistance
TGF-β: Transforming growth factor-beta.
